# Wireless *in-situ* Sensor Network for Agriculture and Water Monitoring on a River Basin Scale in Southern Finland: Evaluation from a Data User’s Perspective

**DOI:** 10.3390/s90402862

**Published:** 2009-04-21

**Authors:** Niina Kotamäki, Sirpa Thessler, Jari Koskiaho, Asko O. Hannukkala, Hanna Huitu, Timo Huttula, Jukka Havento, Markku Järvenpää

**Affiliations:** 1 Finnish Environment Institute, Jyväskylä Office / PO Box 35, FIN-40014 University of Jyväskylä / Finland; E-Mail: timo.huttula@environment.fi; 2 MTT Agrifood Research Finland / Luutnantintie 13, 00410 Helsinki, Finland; E-Mails: sirpa.thessler@mtt.fi; hanna.huitu@mtt.fi; jukka.havento@mtt.fi; markku.jarvenpaa@mtt.fi; 3 Finnish Environment Institute / PO Box 140, FIN-00251 Helsinki, Finland; E-Mail: jari.koskiaho@environment.fi;; 4 MTT Agrifood Research Finland, Plant Protection, R-building, 31600 Jokioinen, Finland; E-Mail: asko.hannukkala@mtt.fi;

**Keywords:** Sensor networks, agriculture, environmental monitoring, data quality, network maintenance

## Abstract

Sensor networks are increasingly being implemented for environmental monitoring and agriculture to provide spatially accurate and continuous environmental information and (near) real-time applications. These networks provide a large amount of data which poses challenges for ensuring data quality and extracting relevant information. In the present paper we describe a river basin scale wireless sensor network for agriculture and water monitoring. The network, called SoilWeather, is unique and the first of this type in Finland. The performance of the network is assessed from the user and maintainer perspectives, concentrating on data quality, network maintenance and applications. The results showed that the SoilWeather network has been functioning in a relatively reliable way, but also that the maintenance and data quality assurance by automatic algorithms and calibration samples requires a lot of effort, especially in continuous water monitoring over large areas. We see great benefits on sensor networks enabling continuous, real-time monitoring, while data quality control and maintenance efforts highlight the need for tight collaboration between sensor and sensor network owners to decrease costs and increase the quality of the sensor data in large scale applications.

## 1. Introduction

The rapid development of sensor and wireless communication technologies has increased the use of automatic (wireless) sensors in environmental monitoring and agriculture [1]. The availability of smarter, smaller and inexpensive sensors measuring a wider range of environmental parameters has enabled continuous-timed monitoring of environment and real-time applications [2,3]. This was not possible earlier, when monitoring was based on water sample collection and laboratory analyses or on automatic sensors wired to field loggers requiring manual data downloading. During the previous decades, environmental monitoring has developed from off-line sensors to real-time, operational sensor networks [4] and to open Sensor Webs. These are based on open, standard protocols, interfaces and web services [5–7].

Varying terminology, such as wireless sensor networks, environmental sensors networks [4] and geo-sensor networks [8], are used interchangeably to describe more or less the same basic concept of collecting, storing and sharing sensor data, but employing different technologies or having different functional focus [2]. All of these terms refer to a system comprised of a set of sensor nodes and a communication system that allows automatic data collection and sharing through internet based databases and services [2,4]. The sensor webs are also seen as an advanced part of sensor networks by some authors [4,8], while others differentiate between sensor networks and sensor webs. They emphasize that the latter are based on open Sensor Web enablement (SWE) standards and web services, and that sensor nodes are able to communicate with each other. This makes sensor webs interoperable and intelligent systems that can react to changing environmental conditions [2,5,6].

Along with developments in sensor and communication technology, complex environmental problems such as eutrophication and climate change have rapidly increased the need for temporally and spatially accurate data [4]. Adaptation to more variable weather and environmental conditions increases the importance of (near) real-time information that is valuable in better timing and control of agricultural management practices such as irrigation and pesticide spraying, monitoring algae bloom, and developing flood and frost warning systems [1,3]. The agricultural and food sector has also faced growing demands for traceability and quality of products in terms of environmental impacts of food production and food safety. This means optimizing cultivation inputs so that high yields are obtained and environmental effects are minimized. Several multinational and national initiatives aiming to improve quality of sea, lake and river water need more accurate information on effective means to decrease contaminants and nutrient discharges to waters and lower their effects, such as cyanobacteria blooms [9–11].

In order to function properly, sensor networks for water monitoring and agriculture normally require a relatively dense deployment of sensors. This leads to applications that monitor mostly local weather and soil characteristics [4]. Agricultural sensor networks have been developed for frost [3] or crop pest warning [12]. They are also an essential component in more advanced decision support systems (DSS) for crop protection [13,14]. In precision agriculture the studies have been concentrated on spatial data collection through mobile, vehicle embedded sensors or *in-situ* sensors deployed in the field [1]. Precision irrigation and fertilization and husbandry monitoring systems based on sensor networks have also been developed [1,15]. In water monitoring sensor networks are used for monitoring water quality and hydrology of rivers, lakes and reservoirs and for flood warning [4,5,16–19].

Although sensor networks still struggle with technical problems, such as energy-consumption, unreliability of network access and standard or software mismatches [20–22], they have already been used for long-term monitoring under harsh outdoor conditions. They allow monitoring remote, hazardous, dangerous or unwired areas, for instance in the monitoring and warning systems for tsunamis, volcanoes, or seismologic phenomena. The sensor webs, in turn, are an emerging technology, that is not yet in operational use outside the test beds [6].

The sensor networks and sensor webs have a profound effect on the collection and analysis of environmental data. The data is very heterogeneous and may come from different *in-situ*, mobile or satellite sensors that have different temporal and spatial resolutions that may vary in accuracy and content [8]. Furthermore, the user has less control over data quality, and information needs to be extracted from a large amount of heterogeneous data. This highlights the importance of comprehensive metadata describing the sensors, data, and data quality, as well as the need for effective tools for data mining or other data gathering [4].

We present here a wireless sensor network (WSN), called SoilWeather, which aims to provide temporally and spatially accurate information, data services and (real-time) applications for water monitoring and agriculture on river basin and farm scales. We evaluate the performance of the network from the data user and network maintainer perspectives, and thus, focus on maintenance and data quality issues as well as applications. The technological development, solutions and standards are already comprehensively discussed in review articles of Yick *et al*. [21] and Akyildiz *et al*. [22]. We also discuss the challenges facing the SoilWeather WSN and the opportunities it has provided. Finally we conclude with the lessons learned from deployment and 1.5 years of running of network.

## 2. SoilWeather sensor network and applications

### 2.1. Karjaanjoki river basin

**Figure 1.**
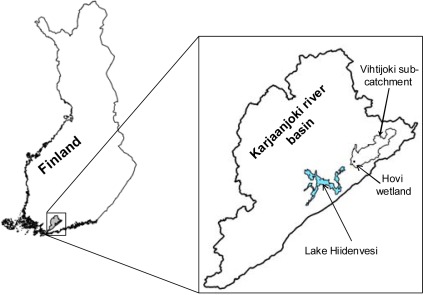
Location of the Karjaanjoki river basin in Finland and the intensive measuring areas of Lake Hiidenvesi, the Hovi farm and the Vihtijoki sub-catchment.

SoilWeather is an operational river basin scale *in-situ* wireless sensor network that provides spatially accurate, near real-time information on weather conditions, soil moisture and water quality with a high temporal resolution all-year round. The network was established in Southern Finland during the years 2007 and 2008 and it covers the entire 2,000 km^2^ Karjaanjoki river basin which is located in south west Finland (Figure 1). The catchment is mainly covered by forest (63%) and agricultural areas (17.7%). In the north part of the area the River Vanjoki and River Vihtijoki bring waters to Lake Hiidenvesi (area 29 km^2^, mean depth 6.7 m) from which waters flow via River Väänteenjoki to Lake Lohjanjärvi (area 92 km^2^, mean depth 12.7 m). Finally, the Mustionjoki river transports water from the river basin to the Gulf of Finland. In the northern parts of the river basin geology is dominated by quartz and feldspar. In the south the bedrock is granite. The soil is mainly clay, silt and glacial till [23].

**Figure 2.**
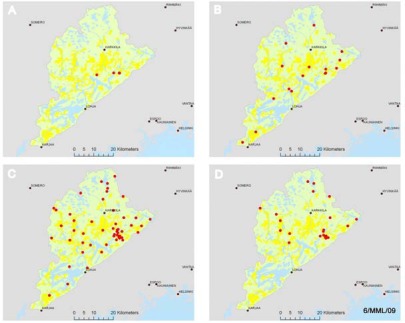
The location of the different SoilWeather WSN stations and sensors in the Karjaanjoki river basin. (a) Nutrient measurement stations. (b) Water turbidity sensors. (c) Weather stations. (d) Soil moisture sensors.

The weather stations are evenly distributed around the catchment (Figure 2). They serve the purposes of catchment wide run off modeling. The turbidity and soil moisture sensors are scattered around the catchment as well, still majority of them are placed on the areas of different applications, which are explained later. Specific nutrient measurement stations are placed totally on the local application areas.

There are three intensively measured areas within the river basin: Hovi farm, Vihtijoki sub-catchment and Lake Hiidenvesi (Figure 1). The sensors are mainly located on land owned by private farmers, who are also the main users of the data. Eleven of the weather stations are placed in or close to potato crops for potato late blight warning. In addition data from one weather station close to a potato late blight control experiment at Jokioinen outside the SoilWeather network was used to evaluate the validity of potato late blight forecasts. The water measurements are obtained mainly in the rivers, but also in relatively small ditches and in constructed wetland within the Hovi intensive measurement area.

**Figure 3.**
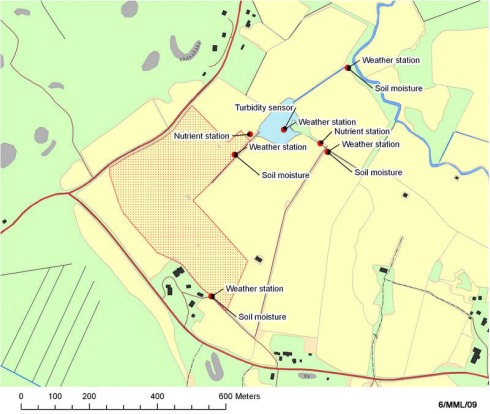
The locations of the SoilWeather WSN's weather stations, soil moisture sensors, nutrient stations and turbidity sensors in the area of Hovi farm.

In the Hovi farm (25 ha) we measured soil moisture, weather and water quality at a field parcel level. The Hovi farm in Vakola is owned by the governmental MTT Agrifood Research Finland research institute. The soils are mainly clay, silt and glacial till and altitudinal variation is low (up to 130 m). Crops include barley, grass, turnip rape and wheat. Constructed wetland was built at Hovi farm in 1998 for water treatment, biodiversity and landscape purposes. The catchment of the wetland (12 ha) is under cultivation. It is a relatively large constructed wetland, ca. 5 % of the whole catchment [24]. One turbidity sensor is installed in the middle of the wetland and two spectrometers measuring nutrient concentration are located in the inflow ditch and close to the mouth of the outflow ditch of the constructed wetland to monitor its effectiveness in nutrient retention. Additionally, there are five weather stations in the area of the Hovi farm. (Figure 3).The spatially dense instrumentation of Hovi enables monitoring and testing of water protection methods and management practises and studying nutrient leaching from agricultural land in varying weather conditions at the field parcel level.

The Vihtijoki sub-catchment, located in the north-west of the Karjaanjoki river basin, is instrumented with 25 weather stations and six water turbidity sensors. The turbidity sensors are located in the upper, middle and lower parts of River Vihtijoki to obtain validation data for the modelling efforts on transport of phosphorus (P) and total suspended solids (TSS) at a catchment level. Here, the SWAT (Soil and Water Assessment Tool) model will be used. SWAT is a catchment-scale model that operates on a daily time step [25, 26] and simulates water and nutrient cycles. SWAT model needs time series of many weather parameters as a part of input data. We intend to test the sensitivity of the model to the frequency of weather stations, i.e. if, and how much, will the results improve when the number of the weather stations will be increased in the model-setup, which will be established in the Vihtijoki sub-catchment (Figure 1).

Lake Hiidenvesi is one of the largest lakes in Southern Finland. It has recreational importance and it serves as a backup drinking water reserve for the inhabitants of the Finnish capital area. Restoration of the lake was started already in 1995 due to low water quality but improvements in water quality have not been gained so far [41]. The SoilWeather WSN has been used to monitor the water quality of the inflow and outflow of the lake using two nutrient measurement stations and one turbidity sensor.

### 2.2. Sensors, sensor network and infrastructure

The Soil Weather WSN hosts 70 sensor nodes altogether; 55 compact weather stations, four nutrient measurement stations, and 11 turbidity measurement stations. Six of the turbidity stations have water level pressure sensors as well. The typical setup of a weather station includes a weather station core and sensors for air temperature, air humidity, precipitation, wind speed and wind direction. Connected to the weather station cores there are also sensors for soil moisture and for water turbidity so that the network observes in its entirety soil moisture in 30 sites, turbidity in 18 sites and water level in eight sites.

Nutrient measurement stations measure water turbidity and nitrate concentration with spectrometers employing ultraviolet and visible (UV-Vis) wavelengths. The setup includes also sensors for water level and temperature. All the sensor nodes have been geo-located in the field using a hand-held GPS device Trimble GeoXT. The sensor nodes, sensors and parameters measured are shown in Table 1.

SoilWeather WSN uses off-the-shelf sensors, nodes and server services provided by various sensor vendors. Each sensor node has a central processing unit with a GSM modem and SIM-card installed either into a weather station core or into a nutrient measurement station. The weather station cores can be controlled remotely by SMS messages or locally by connecting sensor nodes to the computer. The cores can also be programmed to produce automatic SMS alerts e.g. on drought, frost or moisture conditions predisposing to plant diseases.

The network uses time-based data collection. The frequency for nutrient measurements is once every hour, all the other sensors measure once every 15 min. Each sensor node collects and transmits the data independently to the database server, either as a SMS message (a-Weather station cores) or as a data call (nutrient measurement stations). The weather station cores are wireless and automatic; GSM and GPRS techniques are used in the data transfer and storing. GSM modems receive SMS messages, GPRS messages are transferred through HTTP interface. These messages are written to a message database and decoded with a parser program to measurements and timestamps. This information is then written to the final database.

**Figure 4.**
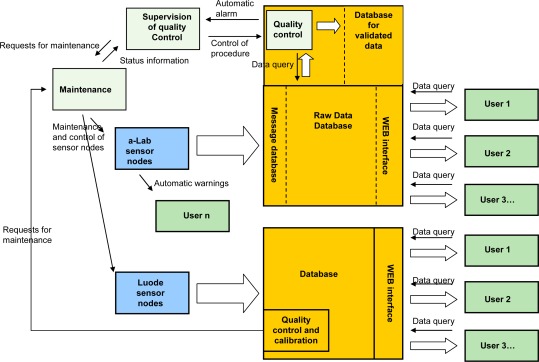
The data flow (white arrows) and communication system with main data services of SoilWeather WSN. a-Lab sensor nodes refer to nodes employing a-Weather station cores, Luode sensor nodes refer to nutrient measuring stations.

The near real-time data is available as graphs and downloadable tables in two different internet-based data services provided by the sensor vendors. One of the services also supports XML-based data transfer. Diagram of the data flow in SoilWeather WSN is presented in Figure 4.

The SoilWeather WSN functions all-year round. Due to freezing of the sensors, measurements are less accurate in cold winter times, as there is no heating in the rain gauges or wind sensors. Also, the sensors located in rivers may be temporally removed during winter, as moving ice might break the sensor probes.

The weather stations are compact devices including all the sensors installed and they are easy to deploy. The weather stations are programmed to connect to the server automatically. For water turbidity and nutrient measurements, the easiness of deployment is very much dependent on environmental conditions, such as the ground material of the river bank and river bed, the river run-off, and existence of constructions. The sensor nodes are transferring data independently, and network is flexible to some extent; it does not demand reprogramming or updating of the existing nodes when new node or sensor is added. The sensor nodes use a battery package of two 6 V batteries.

At the moment, the data for the whole network is available only for participants of the project. The weather measurements are, however, freely available for the previous month through the open interface at the web site http://maasaa.a-log.net/ (in Finnish) and through the web site of Helsinki Testbed (http://testbed.fmi.fi/) after registration to researcher’s interface.

### 2.3. Data quality control and network maintenance

We see data quality as a broad concept including aspects of deployment, maintenance, cleaning, calibration and automatic data quality control algorithms. Careful deployment of sensor probes is the basis for ensuring good data quality. The location of the probe should be representative, considering the parameter measured. Weather stations are located in open and relatively flat areas and water turbidity sensors in the main run-off in location with no nearby discharging ditches or tributaries. The probes are mainly deployed by the same experienced field assistants from nearby MTT Vakola farm and by following sensor specific procedure. However, the final location of the sensor probes was always decided by the application, and negotiations with the land owners. The probes are also located so that they do not hamper cultivation practices or the recreational use of the river.

All the water and soil sensors are calibrated against water or soil samples, respectively. For weather stations no calibration in the field is done. Calibration samples for water measurements are taken once a month to ensure the quality of the sensor measurements and the correct functioning of the sensors. River discharges are available close to the location of the water measurements. Soil moisture calibration samples were taken soon after the deployment.

Reliable functioning of the sensors requires maintenance often enough. We maintain sensors on a regular basis, twice a year, but also occasionally when additional maintenance is needed. The maintenance procedure is sensor type specific. For weather stations the batteries are changed once a year, the fixation of instruments is checked and fixed if needed, and the equipment is cleaned. The water turbidity sensors and nutrient measurement stations need extra care because the optical lenses get contaminated in the water. The spectrometers are cleaned automatically with air-pressure and in addition manually once a month. Some of the water turbidity sensors are equipped with automatic wipers. The wipers were not available during the first deployments so the sensors were manually cleaned in regular basis: in winter time every month and in summer time when needed, approximately once a week.

**Figure 5.**
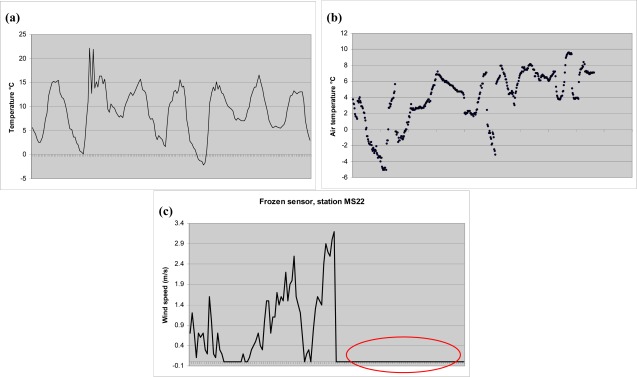
Problems that have occurred in SoilWeather WSN data. Y-axis denotes different parameters and the x-axis denotes time (approx. 1 week). (a) Suspicious spike in air temperature data. (b) Gaps in the air temperature data. (c) Wind speed is constantly 0 m/s (no variation).

Automatic data quality control system, that warns when suspicious data is received, was developed during the project to notify on maintenance needs. Different kinds of data quality problems that can occur in the SoilWeather WSN data are shown in Figure 5. In the first chart (a) there are two suspicious spikes in the temperature data. Rather common situation of missing data is shown in the second chart (b) and in the third chart (c) the wind speed is for some reason measuring the same value (0 m/s) all the time. In the beginning of the project there were only a few stations providing data to be checked and the quality control was carried out manually. As the amount of stations, and therefore the amount of the data, grew, it was essential to develop an automatic quality control and warning system. At the moment the system checks the data from all the a-Lab sensor nodes. For the four nutrient measurement stations Luode Consulting handles the quality control manually using their strong expertise and experience in this field.

The automatic quality control runs under the UNIX system. The computer of the data quality controller logs in to the a-Lab server via SSH tunnel and retrieves data using Matlab Database Toolbox. After the tests are run in Matlab, the quality controlled data is returned to a new database in a-Lab server. At the moment there are four different tests running in near real-time:

1. missing data test
2. missing observations test
3. variation test and
4. range test.

The missing data test checks if the data has been sent correctly. If no observations have come from the sensor within the period after the last check, the system saves an error report. Meanwhile the missing data test checks long periods of missing data, the second test searches for occasional missing values. The third test is for checking if the measurements vary over time. Presumably there is something wrong with the station or the sensor if the sensor measures the same value consistently (for 24 hours in this case). Finally, the range test tests if the measurement lies between predetermined range values. For meteorological parameters limit values were configured based on seasonal climate extremes and limit values vary according to the month and the climatic zone. The climatic zone of the Karjaanjoki river basin is hemiboreal and the range values in this case for air temperature are shown in Table 2. Limit values for meteorological parameters are provided by the Finnish Meteorological Institute (FMI). Soil humidity, turbidity and water level ranges are defined for every sensor separately, depending on the characteristics of soil, riverbed and river hydrology. For every observation the system gives an information label (flag) that indicates the quality level of the observation according to the range test. The flag value indicates whether the observation is correct (between the range values), suspicious (differs slightly from the range value) or wrong (differs dramatically from the range value). The range test and the flagging follows the system used in FMI [28].

All the error messages from the past 24 hours are collected and sent automatically by e-mail to the data controller every morning. After the notification, the controller checks the data manually and makes the decision weather to inform the maintenance team or not. All the maintenance and the cleaning activities are stored in the log file of the sensor node and the log file is available for users through the data services.

### 2.4. Applications

SoilWeather WSN is designed to be a multi-functional network. During the two-year pilot project, it has been utilised in the following applications:

- in predicting potato late blight risk
- in developing interpolation methods for weather parameters into 30 m resolution grid
- in monitoring water quality and nutrient retention in rivers and in constructed wetland
- in improving hydrological model at river basin scale
- in leaching model in sub-catchment scale
- in soil moisture model at field parcel level
- in precision agriculture.

It has also been used to study the relationship between local weather conditions and nutrient leaching. The network enables monitoring weather-related phenomena, such as heavy rains and the nutrient load peaks they induce. The SoilWeather WSN is used in research and in governmental monitoring tasks, but also by private farmers, who can use local data in planning and executing management practices. Here we present and analyze two applications in detail: predicting potato late blight risk in the farms, and the monitoring of constructed wetland.

**Figure 6.**
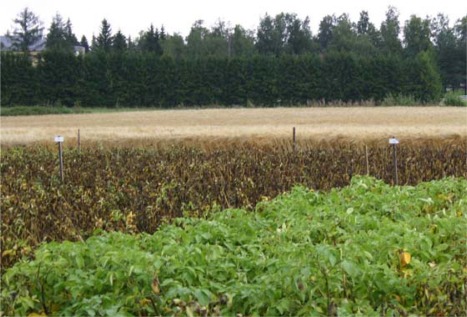
Potato late blight can totally destroy potato crop. Consecutive fungicide applications (green area in the front) are needed for effective control of blight. (Photo: MTT.)

Potato late blight caused by an oomycete, *Phytophthora infestans*, is one of the most devastating potato diseases worldwide. The potato crop can be completely destroyed within a few days if the weather is conducive for disease progress (Figure 6). In modern conventional potato production late blight can be effectively controlled with a range of chemical fungicides. The potato crop must be protected from emergence to harvest for each single day when weather enables late blight infection. Fungicide applications are necessary at 3 – 10 days intervals throughout the growing season resulting in 4 – 10 consecutive sprays in Nordic production and more than 20 sprays in the most intensive potato production regions in Western Europe [29, 30].

To optimize the number of fungicide applications per season numerous weather based blight forecast models have been developed since the 1950s [31]. In the Nordic countries a late blight forecast model (NegFry) developed by Fry *et al*. [32] has been widely used since the 1990s [13]. Dramatic changes in the epidemiology of potato late blight pathogen have made the old NegFry model unreliable in certain occasions [33, 31]. Therefore a more recent potato late blight model (LB2004) introduced by Andrade-Piedra *et al.* [34] has been modified for use in the Nordic climate [35]. The characteristics of current Nordic potato late blight populations for the model development were studied in detail [36] and essential epidemiological parameters needed in the model were updated [37]. Sub-model calculation periods when the temperature is over 8 °C and relative humidity is over 90 % [35] was used to predict blight risk in this study.

The blight risk was calculated for the 11 weather stations at the potato fields and at the weather station at late blight control experiment at Jokioinen. The potato fields were visited twice a week from the last week of June to the first week of August. The occurrence of potato late blight was recorded and the onsets of blight epidemics were reported in the Web-Blight warning service (www.web-blight.net). The severity of blight as a percentage of defoliated leaf area was assessed at the experiment at Jokioinen three times a week.

Constructed wetland studies were made during 1999–2002 at the previously mentioned Hovi wetland [24]. As for the monitoring of water quality of inflow and outflow, the measurements were based on water sampling. Although the sampling earlier was flow-proportional and rather frequent, most of the days were left unmonitored. However, these days may include short-termed peaks of high runoff, which remain unknown. Typically, the gaps between the sampling days have been filled by e.g. linear interpolation, but the loading estimates tend to be more or less erroneous. Flow variations and thus also the error is particularly significant in small, agricultural, high-sloped catchments like the Hovi farm. For this defect, automatic sensors providing non-interrupted data offer a revolutionary improvement. To test this new monitoring approach in wetland research, s::can -sensors (Table 1) were installed in October 2007 for monitoring of the water entering and exiting the Hovi wetland at 1-hour interval. The first full 1-year results (from November 2007 through October 2008) on the retention performance of the wetland were compared with the previous, water-sampling –based results [38].

## 3. Results and discussion

### 3.1. Performance of the network

Several authors have discussed the reliability problems of WSNs. However, reliability is normally discussed from a technological perspective. Thus, diagnostic and debugging as well as communication protocols are analyzed in relation to the application and power-consumption [1, 21, 22]. These studies are important for recognizing missing measurements due to unreliable communication or sensor failure, but normally are unable to identify erroneous measurements. Sensor calibration and means to recognize and discard data from wrongly calibrated sensors has been also important aspect in ensuring data quality [8, 4].

We analyze the performance of SoilWeather WSN by analyzing both missing and erroneous measurements, as well as the maintenance needed. The number and types of the maintenance visits are clarified and the problems with certain sensors are examined. The performance of the quality control is estimated by analyzing the number of erroneous and missing measurements. The better quality of the turbidity data is ensured by installing automatic cleaning wipers. The performance of turbidity wipers is analyzed by comparing turbidity values before and after the installation.

There are several factors related to the communication network, the stations or sensors themselves and the outdoor conditions that can interfere with the data. The improper functioning of the communication network can obstruct the data transfer and battery consumption. Under normal circumstances, the batteries should function properly for almost a year. However the strength of the signal within the GSM network can affect the battery age: a weak signal consumes more power from the station than a strong signal. Communication network problems can be seen as missing data or delayed data delivery.

Different problems can occur depending on the location of the station, weather conditions, nearby forest stand or the characteristics of the river. For example rain gauges tend to fill with leaves, tree needles and bird droppings thus distorting the precipitation data. In winter time the turbidity sensors might get broken due to moving ice. The first winter of the project was warmer than usually allowing the turbidity sensors to stay in the water for the whole winter without problems of freezing. In normal winter (as the second winter was) majority of the turbidity sensors has to be picked up for the coldest months.

**Figure 7.**
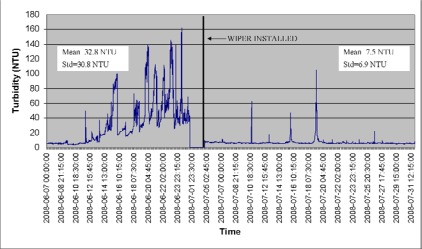
An example of the effect of a turbidity wiper installed to one of the SoilWeather WSN's turbidity sensors. Turbidity measures a month before and after the wiper has been installed. The mean and standard deviation before and after installation.

Another problem concerning the turbidity measurements is the bio fouling of the optical lenses. Especially in summer time this is a big problem and the sensors would require cleaning on a regular basis, even daily in the most turbid waters. Also water plants, fish, gastropods or other objects in the water may affect the sensors. During the project we have discovered that almost without exception all the turbidity sensors need some kind of automatic cleaning system. At this point the wipers have been installed on the six sensors that have had most problems with biofouling or on sensors that are a long way from the MTT Vakola farm. The drastic effect of a wiper installed to a place that has normally very turbid water can be seen in Figure 7.

After the wiper has been installed, the level of turbidity has decreased dramatically. In addition there is no sign of the growing trend caused by the gradual contamination of the sensor. The real increase and level of the turbidity can now be seen in the data. The single spikes still remain in the data as they are caused by occasional disturbances. Obviously the mean value and standard deviation have decreased significantly in the study period. The cleaning of the turbidity sensors has probably been the most laborious maintenance task during the project. Furthermore the contamination of the turbidity sensors has caused most drastic errors to the data. With the help of the wipers these problems can partly be overcome.

In addition to maintenance of the turbidity sensors, battery problems have been quite major ones as well. Before the right composition of the battery package had been discovered, the gaps in the data were due to battery voltage decreasing or problems with battery contact. There have been five mysterious malfunctions of the stations as well. These stations had to be sent back to vendor and wholly repaired and reprogrammed. The main reasons for maintenance, how they occur in the data and how many occasions there has been during 1.5 years period are shown in Table 3.

The automatic quality control and warning system developed for detecting the most drastic errors has worked relatively well. Here we analyze the suspicious and erroneous measurements for period of four months, from July 2008 to October 2008. Only very small fraction (0.06 %) of the measurements was outside the range of the limit values. The total numbers of suspicious and erroneous measurements defined by the range test are shown in Table 4.

Air temperature was over the range limits for one station in one occasion due to few cold summer nights as the temperature decreased under the range value of 0 °C. Air pressure measurements have been below threshold value because of the decreasing battery voltage. In this case the problem was detected and solved fairly quickly. There have been 900 suspicious and 30 erroneous water level measurements. One of the water level sensors was for some reason pulled to the shore and another one was installed in a place whose water level decreased so much that it had to be moved to another spot. The total number of suspicious and erroneous turbidity measurements was ca. 400. These (mostly too high) turbidity measurements were caused by single spikes in the data.

In general the sensor nodes and data transfer have been working well with regards to the missing values. At this point we only report the total number of problem occasions. Detailed analysis of lost data and their time span will follow later. For the weather stations the median of the proportion of the missing values was 0.6 % and for turbidity measurement stations 1.4 %. Due to different problems described earlier the variation was quite high: for some stations there have been missing values for over 10 % of the measurements. These missing values have usually been due to battery problems and therefore we have not found any differences according to the latitude component for example.

For good network functioning, it is essential that we are informed as soon as possible if some of the sensors or stations are not working at all. Altogether we still see the need to develop further the data checks and this way reach an optimal data flow and quality of the data. The present ability to detect the most obvious problems is a good start for this.

### 3.2. Performance of applications

Weather stations at the potato fields have been functioning relatively well. In the beginning of the season there were some technical errors in the measurement of relative humidity at some stations. The problems were solved and correct measurements were obtained during the critical period for potato late blight development. Blight risk at all potato fields was low until 9^th^ of July. Between 10^th^ and 25^th^ of July there were 10 – 15 days when the temperature was over 8 °C and the relative humidity over 90 % for more than 10 hours. Blight risk was low from 26^th^ July to 2^nd^ August. From 3^rd^ of August blight risk was very high until the end of August.

Blight was found at one field at the beginning of the high risk period 12^th^ of July. After the high risk period the late blight was present in all fields at the end of July. During August the disease spread rapidly causing severe damage to three fields, where fungicide applications were started too late. In the rest of the fields blight was adequately controlled by fungicide applications. Blight risk estimated by duration of moist periods was relatively well in line with the observed blight epidemics.

**Figure 8.**
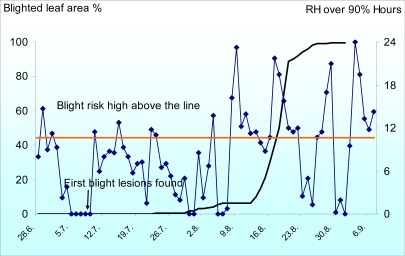
Duration of periods (hours), when relative humidity was more than 90 % and temperature over 8 °C and progress of potato late blight epidemic (percentage of defoliated leaf area) at Jokioinen in non-protected susceptible potato cultivar in 2008.

In the field trial at Jokioinen blight progress was very similar to the potato fields at the SoilWeather network. Blight was found the 11^th^ of July but the epidemic developed very slowly until August due to rather low blight risk. An epidemic exploded in the middle of August after several successive days when relative humidity was over 90 % more than 10 hours (Figure 8).

In wetland monitoring the turbidity sensors and the weather stations were used. In order to be accurate and reliable, automatic monitoring with sensors needs water samples for calibration of sensors for the specific conditions of the measurement place. Moreover, concentrations of some important substances –such as dissolved P– can not be measured with commonly available sensors thus leaving the laboratory analyses of the sampled water as the only option. In this study, a total of 75 samples were taken from the inflow water (24 manually and 51 with a refrigerator-equipped sampler). As for the outflow, 21 samples were taken manually during the 1-year study period. All water samples were analyzed for turbidity and the concentrations of TSS, total P (TP), dissolved reactive P (DRP) and nitrate (with nitrite).

The s::can sensors deployed in the Hovi wetland were calibrated using linear regression equations between the sample-based values and the simultaneous recordings of the sensors (“raw data”). Each recording of the raw data was then multiplied with the coefficient obtained by the regression equation of respective substance (turbidity or nitrate). Such calibrated values were used in the calculations of material fluxes. The final data curves were found to correspond well the sampled values [38], which suggested that the sensors functioned reliably.

Because turbidity does not represent an amount of substance in water it can not, like nitrate concentration, be directly used in the calculation of material fluxes. Fortunately, in the case of Hovi, correlations between turbidity and the concentrations of TSS and TP were very high with a coefficient of determination (R^2^) of 0.86 or more. Thus, we could reliably transform the calibrated turbidity values into TSS and TP concentrations by multiplying them with the coefficients obtained from the linear regressions.

TSS and TP retentions in wetland (70 and 67%, respectively) were at a similar or slightly higher level than in the previous measurements. Meanwhile the value for nitrate retention (67%) was strongly increased. The improved nitrate retention suggests the positive effect of the vigorously expanded vegetation during the unmonitored time between the two study periods in the Hovi wetland.

Wetland measurements with sensors have been thus far successful. The information obtained with new technology has not only provided more accurate retention figures, but also given new insight on the behavior of TSS and nitrogen in a CW.

### 3.3. Benefits and challenges

**Figure 9.**
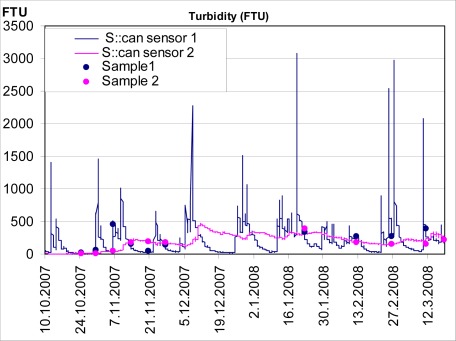
Turbidity samples (dots) and turbidity measured by spectrometer (solid line) from the constructed wetland of Hovi.

Due to the high temporal resolution of the measurements, SoilWeather WSN provides significantly more accurate information on nutrient leaching in different weather conditions at parcel level than can be achieved by regular sampling. Figure 9 shows turbidity measured with the spectrometer. The turbidity measured from water samples during the same time period indicates that most of the nitrate leaching peaks remains unnoticed highlighting the efficiency of sensor networks to monitor irregular, short events. The in-situ sensor networks provide point measurements that can be used as input in leaching models and, thus, improve the estimates of nutrient leaching at different scales.

The potato late blight forecast can be applied to justify precise timing of fungicide applications. In the future, forecasts for other crops and pests should be developed. Relatively good models that predict *Sclerotinia* diseases of oil seed crops exist [39]. More effort is needed to develop applications for cereal diseases while there is clear demand for such forecasts among farmers and advisors.

On the other hand, as the SoilWeather WSN provides over 30,000 measurements per day and data accumulates progressively over time, this poses significant data processing challenges. Due to the large amount of data erroneous or missing measurements need to be tracked and when possible, also corrected by automatic algorithms. Protocols for error diagnostics and debugging of WSNs have been developed that notify when measurements are missing due to communication, device or software faults [21, 40]. Determination on erroneous measurements, in turn, is sensor and environment specific, and base on statistical calculation and regular calibration samples.

Good sensor data quality is a critical factor for data users. Evolving standardising and increasing joint use of sensor data has been seen to lead to unforeseen data availability in the future [4]. It is also more and more critical for data user combining different data sources to be informed of the quality of data by providing quality estimates for measurements and documenting data quality and the control procedure. Joint use of sensor networks and webs requires development of open standard protocols and interfaces as well as open source software products to discover and analyse sensor data from different sources [7, 4]. The internet based data services enable easy access to data and metadata by users and applications in the case of single sensor network. At the moment SoilWeather data is collected in two different servers and respectively in two web services provided by sensor vendors used. In addition, the download of the data tables needs to be done station by station. In the next phase of SoilWeather WSN, it is important to develop web services so that they support easy data access and use. Also data flows from sensors and calibration samples need to be integrated and easily available.

If the large amount of data poses challenges to the user of sensor networks, the maintenance of the large sensor network is a challenge for the data provider. The monitoring over a large area and especially the continuous sensing of water requires maintenance resources. The amount of the field work and maintenance costs may be the same as in field logger based sensing. The sensors measuring water quality need regular cleaning in the field and calibration samples need to be taken. Depending on the locations of the sensor nodes, one field worker can maintain 5–10 sensors per day. Total maintenance costs of the whole SoilWeather network including also laboratory analyses of water and soil samples, data transfer costs, and costs of replacement parts and batteries are not available yet. Still they should not be underestimated, because it plays a key role in ensuring the quality of sensor data.

Major strength of the SoilWeather WSN is the tight collaboration between three governmental research institutes, Agrifood Research Finland, the Finnish Environment Institute and the Finnish Meteorological Institute. This way we have been able to establish a multipurpose network requiring a wide range of expertise and to develop a wide range of potential applications. The network is also established in collaboration with private enterprises including sensor vendors, data users and service developers. The low costs of the weather stations make it possible for individuals, such as farmers, and for small organizations to participate in the sensor networks in the future. Admittedly, data provided by the network would be interesting for other sectors such as tourism and traffic as well.

Considering the maintenance efforts, increased collaboration, open standard protocols and interfaces are seen as being important in the future development of SoilWeather WSN. By collaboration it is possible to create a cost-effective monitoring system that covers wide areas, provides data of good quality from different types of sensors and encourages joint use of data. Maintenance costs are decreased if the work is done close to the sensor location, whereas synergy is obtained if data quality procedures and algorithms are defined and developed, and employed together over the large group of data providers. When the number of data providers becomes larger, the control over data quality decreases. Therefore it is important to ensure that the data collection, processing and data quality is well documented and delivered to the users.

However, this collaboration requires open and widely used technology and standards that enable integration of different sensors and sensor data and flexible integration of new sensor nodes as well as tools for storing, archiving and delivering of data. The Sensor web enablement (SWE) of Open Geospatial Consortium (OGC) provides the needed standards and tools. It is also increasingly tested in a range of applications from earth observation through satellites to delivery of hydrological data [7]. If this technology shifts to operative use, it would also enable the discovery and exchange of data on a larger scale, through organizations, sectors and countries.

In addition to challenges in data processing and field maintenance we see data sensitivity and the attitudes of data providers as a third challenge. Especially if data is to be made freely available through web services. In the SoilWeather network, data on water quality is available only for the participants of WSN, while the weather data is already publicly available. This is because we want first to ensure the good quality of water measurement data by fully operational quality control procedures. However, aquatic measurements are also considered more sensitive data than meteorological data. In the case of SoilWeather WSN, farmers often do not want nutrient leaching rates available for anybody to follow if there is even a minor risk that high rates could cause changes to the management practices of the farm. One should, however, notice that the aims of the authorities and farmers are more or less congruent: both of them benefit from low nutrient leaching to the rivers. Thus, it is also a question of attitudes that hinder collaborative monitoring of water quality. The water quality data is also sensitive from the perspectives of other potential private data providers than farmers. These could be enterprises providing drinking water for example. The public sector, in turn, is already opening their large environmental databases and supporting joint use of data. For example, the Finnish Environment Institute has recently opened a web service, Oiva, which provides most of the hydrological and water quality data collected by the environmental authorities in Finland (http://www.ymparisto.fi/oiva, in Finnish).

## 4. Conclusions

The SoilWeather network is still in the initial phase of the operation. Thus, not all the maintenance and sensor calibration procedures (particularly for soil) are fixed and data quality control algorithms for water and soil measurements are under the development. It is already clear that relatively high maintenance resources and effective data quality control are needed. However the maintenance efforts of the SoilWeather WSN can be decreased to some extent by the efficient organization of work, with good collaboration and by technical development of sensors and automatic cleaning systems. The amount of field work needed in aquatic data collection is higher than in meteorological and terrestrial data collection and the amount of fieldwork needed is expected to be no less than on the conventional, sampling based monitoring.

At the moment, the automatic quality control tests run on the SoilWeather WSN reveal the most drastic errors in the data, and warn of missing data. However, there is a need for more sensitive tests. Tests that compare the values of neighboring stations would be effective in detecting the faults in precipitation data for example. Consistency tests, on the other hand, would test if different parameter values of the same station are physically and climatologically consistent. For example the values of turbidity and precipitation or turbidity and water level depend on each other.

In the future development of SoilWeather WSN, we have to address three major challenges: 1) a large amount of data, 2) cost-effective maintenance of WSN and 3) the sensitivity of the data and the attitudes of data-owners towards data sharing. These challenges from user and data provider perspectives need to be considered when building operational environmental monitoring systems over a large area. However, we see the benefits of continuous environmental monitoring, and the increased accuracy of provided data, large enough to motivate overcoming the challenges. Furthermore, challenges may be partly overcome by good collaboration and development of tools for data quality control and data processing. To ensure the quality of data and decrease the heterogeneity of measurements, there is a need for handbook for WSN data providers on how to carry out automatic monitoring of environment, particularly related to the water measurements.

SoilWeather WSN is currently functioning and funded on a project basis. A great challenge will then be to find sustainable longer term funding. The obvious options are governmental funding or funding through beneficial business models that are developed on the WSN.

## Figures and Tables

**Table 1.** The sensors used and the parameters measured in the SoilWeather WSN.

**Sensor node**	**Sensors**	**Parameters**	**Producer's web page**

a-Weather station basic core	Pt1000	Temperature	www.a-lab.fi
	AST2 Vaisala HMP50	Humidity	www.vaisala.com
	Davis Rain Collector II	Precipitation	www.davisnet.com
	Davis Anemometer	Wind direction	
	Davis Anemometer	Wind speed	

Additional parameters	Decagon ECHO (capacitance)	Soil moisture	www.decagon.com
	FDR (Frequency Domain Reflectometry)	Soil moisture	www.a-lab.fi
	OBS3+	Water turbidity	www.d-a-instruments.com/
	Keller 0.25 bar	Water level	www.keller-druck.ch

Nutrient measurement station	s::can spectrometer	Nitrate conc.	www.s-can.at/
		Water turbidity	
		Water level	
		Water temperature	

**Table 2.** The monthly range limits (°C) for air temperature in hemiboreal climatic zone. Warning_low and warning_high denotes the range limits for suspicious values, error_low and error_high the range limits for meteorologically impossible values.

**MONTH**	**WARNING_LOW**	**WARNING_HIGH**	**ERROR_LOW**	**ERROR_HIGH**
1	−37	11	−47	17
2	−35	11	−45	17
3	−31	15	−41	22
4	−19	23	−29	27
5	−7	29	−17	31
6	−2	32	−12	36
7	2	33	−8	36
8	0	32	−10	36
9	−7	26	−17	31
10	−16	19	−26	28
11	−23	12	−33	20
12	−35	10	−45	16

**Table 3.** Maintenance intensity of SoilWeather WSN during 6/2007–12/2008.

**Problem**	**Manifestation in the data**	**Number of occasions**

1. Battery voltage decreasing	Missing data and/or air humidity decreases too much	48
2. Turbidity sensor contaminated	Turbidity values too high and increasing	64
3. Problems with battery contact	Missing data or station down	Ca. 40
4. Organisms on the turbidity sensor	Saw tooth pattern in the data	several
5. Rain gauge clogged up	No accumulation of the precipitation despite of the nearby rain	18
6. Station fell down	Possible problems with wind data and/or no precipitation	12
7. Station malfunction	Missing data or station down regardless of battery condition	5

**Table 4.** Number of the suspicious and erroneous measurements by range test during 7/2008–10/2008.

**Parameter**	**Number of suspicious measurements**	**Number of erroneous measurements**

Air temperature	114	-
Water level	900	30
Air pressure	250	-
Turbidity	323	80
